# The general public’s attitude towards accepting payment for kidney donation

**DOI:** 10.3389/fmed.2023.1282065

**Published:** 2023-12-13

**Authors:** Limor Dina Gonen, Ya’arit Bokek-Cohen, Mahdi Tarabeih

**Affiliations:** ^1^Department of Economics, Ariel University, Ariel, Israel; ^2^School of Psychology, Tel Aviv University, Tel Aviv, Israel; ^3^School of Nursing, The Academic College of Tel Aviv-Yaffo, Tel Aviv, Israel

**Keywords:** kidney transplantation, end-stage kidney disease (ESKD), willingness to accept (WTA), cost benefit analysis (CBA), contingent valuation (CV)

## Abstract

**Introduction:**

Kidney transplantation has become the most cost-effective treatment for patients with end-stage kidney disease (ESKD) and offers them the highest quality of life. Yet, kidney donation is often inaccessible due to cultural and traditional beliefs about organ donation. The goal of our study is to assess the value of kidney donation using the Willingness to Accept (WTA) technique. We also aim to understand the factors influencing an individual’s willingness to donate an organ.

**Methods:**

A self-administered survey was completed by 985 participants from the general public. The quantitative method and survey design that were chosen used descriptive, correlational, nonparametric, and multivariate statistical tests.

**Results:**

Most of the respondents, 895 (90.9%) are not willing to donate a kidney while alive. Four hundred and five (41.1%) of the respondents are not willing to donate a kidney after their death, while the rest are willing to donate their kidney after their death without financial compensation. The same attitude applies to the donation of a kidney from their relatives. Significant predictors from the results of the logistic regression model in predicting the lowest (minimal) amount that will encourage donation of one kidney after death were: Marital status; Nationality; Adi card holder; Knowing people who need a kidney donation; confidence in the medical staff; and consideration of the family’s opinions regarding organ donation.

**Discussion:**

Using cost benefit analysis (CBA), with the aim of evaluating the willingness of individuals to accept payment for innovative medical procedures, such as kidney donation, allows an assessment of the perceived value of the medical procedure and enables policymakers to decide whether to allocate funds or offer subsidies for kidney donation, given the limited healthcare resources available. During our research, we found that most participants did not support the commercialization of organs. Our recommendation for policymakers and health professionals is to continue providing adequate funding for kidney donations and to implement educational programs aimed at improving attitudes towards organ donation.

## 1 Introduction

The option of live kidney transplantation is becoming more common for those with end-stage kidney disease (1). Over the past decade, despite implementing several strategies to increase the number of available organs for transplantation the demand for organs still far exceeds the supply. Consequently, the idea of introducing monetary compensation for organ procurement from both living and deceased donors has gained significant attention and reignited the debate in the medical community (2–4). The topic of compensation for living kidney donation is a controversial subject among healthcare professionals and the public (5–9). Unethical inducements in organ donation refer to actions that exploit the vulnerability of potential donors, undermine their autonomy, and encourage monetary incentives to supersede altruistic motives and the commodification of the human body (10–14). Philosophical ethicists and social scientists oppose the selling of organs without regulation. A regulated market can increase the availability of human organs, cut back on transplant tourism, black markets in organs, and exploitation (6, 15–22), and maintain ethical principles such as beneficence, non-maleficence, autonomy, and justice. Rewarded compensation usually refers to various types of monetary incentives to encourage donation; these include tax benefits, paying university tuition, healthcare benefits, offering life insurance packages, and compensating the estate of the deceased donors (23–26). Offering incentives would reduce donors’ danger of health risks, ensure a more stable supply of organs, promote healthier habits for donors and recipients, and provide more medical screening for prospective donors (19).

In Israel, kidney transplantation is not tradable in the free market and is fully funded by the state (27, 28). Israel has implemented a distinctive system of incentives to encourage individuals to register as organ donors and donate organs of deceased first-degree family members. The incentive program provides priority allocation for organ transplantation to living donors should they require an organ donation in the future, as well as to first-degree family members of a potential donor, although they did not personally register as donors. Israel’s innovative approach has proven to be an effective way to increase the number of organ donors and is noteworthy for prioritizing allocation to first-degree family members of potential donors (29).

### 1.1 Research objectives

Our study aims to gain insights into the public’s perceptions, values, and attitudes towards kidney donation. By contributing to the existing body of research, we hope to shed light on the challenges and opportunities associated with kidney donation. To achieve this, we conducted a survey using the CV payment card (PC) technique, applying the Willingness to Accept (WTA) technique. WTA is widely applied in the field of healthcare services; its advantage is in allowing for an evaluation of individual preference to be derived from respondents’ answers.

Based on samples of participants from the general public, through an empirical model, we conducted:

1. Monetary assessment of kidney donation:

1.1 Assessment of the minimum amount that a respondent is willing to accept for a kidney donation while still alive.
1.2 Assessment of the minimum amount that a respondent is willing to accept for a kidney donation after his death in exchange for payment to a family member.
1.3 Assessment of the minimum amount that a respondent is willing to accept for a kidney donation of one of his first-degree relatives (child/brother/spouse/mother/father) after their death.

2. Evaluation of the demographic and complementary predictors of the willingness to accept payment for a donated kidney from: (1) a living donor and (2) a deceased donor.

## 2 Literature review

The organ shortage crisis has been the subject of intense debate on how to encourage legal and ethical organ donation (5–9). The literature discusses the gift versus market dichotomy and altruistic donation versus the market economy (29–33). To address the shortage of donated organs, a third approach has been proposed, which involves a regulated system that offers incentives for organ donation, combining the gift/market concepts (6, 15–22, 32, 34). In this system, the state actively encourages organ donation and compensates donors, recognizing that compensation complements altruism, which remains a crucial component of this socially beneficial act. Instead of making human organs a tradable good by offering them for sale, the state provides incentives to donors as a token of gratitude and appreciation for their willingness to benefit others in need (34). This approach may also help promote a change in societal attitudes and behavior (35–38). Public health policy employs different tools to bring about changes, including legislation, general information campaigns, and positive or negative incentives (29, 39, 40). Incentives for organ donation registration can be categorized as ex-ante incentives offered to potential donors during their lifetime or ex-post incentives offered to family members for consent to donate organs after a relative’s death (19, 23, 24). Incentives can be classified into three categories: non-financial, indirect financial, and direct financial. Non-financial incentives involve granting priority to registered donors if they need an organ (29). Indirect financial incentives provide a symbolic reward for declaring one’s willingness to donate an organ and may include tax benefits, reduced rates on health insurance policies, and bearing part of funeral costs (29, 41). Allocation priority is a public policy incentive adopted by very few countries, with Israel being one of the few that offer this incentive to registered donors (29). Direct financial incentives can be likened to a ‘futures market’ for organ donation after death, where a legally binding contract is signed by the organ donor-seller with the state, which is the only legally authorized client for such a transaction. Upon the seller’s death, the state is authorized to procure the organs for a price that was determined in advance by the regulatory framework and is paid to the seller’s estate or beneficiaries (29). Another direct financial incentive for deceased and living donation is a “regulated organ market.” In this market, the state is the sole authorized purchaser (“single buyer concept”) that buys organs for a fixed price from willing sellers. The sellers are either close relatives in the case of post-mortem donation or the living donor (or seller) in the case of living donation. This incentive involves a “spot market” instead of a “futures market.” Iran is the only country in the world to legalize a free market of kidneys from living donors, a policy that eliminated Iran’s waiting list for kidney recipients (24, 42).

In Israel, the state fully funds kidney transplantation for its residents. Since 1995, the National Health Insurance Law has been in place to regulate the healthcare rights of residents, the basket of health services, and various types of supplemental insurance. Under this law, the state has responsibility for ensuring the health of all residents which is implemented through a basket of health services determined by a government committee. Renal Replacement Therapy (RRT) is considered a “Severe Disease” and is funded differently. With this law in place, every Israeli resident who in need of RRT is entitled to receive it without any charge, irrespective of their socioeconomic status (27, 28). There are currently six medical centers in Israel that carry out organ transplantations. The Israel National Transplant Center (INTC), a division of the Ministry of Health established in 1994, manages all aspects related to donors and organ allocation. Its objectives include promoting organ transplantation, maintaining a centralized record of potential transplant candidates, determining criteria for selecting recipients, proposing guidelines for recipient selection, and collecting data. In 2008 the Israeli Knesset passed The Organ Transplant Law, which delineates the conditions for conducting transplantations from both living and deceased donors in Israel and foreign countries (43, 44). The law also has strict regulations in place to prevent organ trafficking as set forth in the Declaration of Istanbul (45). Despite Israel’s advanced technology and organized healthcare system, the rate of deceased organ donation is lower there than in most Western countries. This is due to the multicultural society of Israel, which comprises various religious groups (Jews, Muslims, Christians, and Druze) and ethnic groups (46). Many Muslim believers have religious and cultural objections to donating organs (47). There is a controversy among Jewish religious authorities regarding the definition of death (48). In 2008, the Israeli Knesset passed the Cerebro-Respiratory Death Act, constituting agreement between the medical community and the religious authorities in defining the criteria for determining brain death but only recently, more rabbinical leaders started gradually to accept the medical definition of brain death and support organ donation as a commendable deed. Nevertheless, there has been no significant increase in the actual number of deceased donors (48). Another reason proposed for the low rate of organ donation is the “free rider” issue (that is, people who object to posthumous organ donation but would apply as candidates to receive an organ if the need arose) which has caused resentment towards organ donation among certain groups. To address this, the Knesset enacted an amended Organ Transplant Law in 2013 that outlines three levels of priority for transplant candidates based on their previous donation history or registration as a donor. Under this law, living donors are given priority in receiving organ donations if they ever need it. Israel also prioritizes the first-degree family members of prospective donors, regardless of whether the former personally registered as donors (49). The Organ Transplant Law introduced additional incentives to promote live organ donation. Donors can receive reimbursement for up to 40 days of lost earnings based on their average income over the three-month pre-donation period, as well as travel expenses to and from the hospital, and a refund for seven days of convalescence in a recuperation facility. Additionally, medical needs, earning loss, life insurance, and psychological consultation for up to five years are covered. These incentives have resulted in a notable increase in live kidney donations (43). The INTC is currently working to encourage organ donation after cardiovascular death. This includes conducting informative workshops on organ donation for Jewish and Muslim religious leaders and enlisting the support of a Knesset advocacy group comprising members from both communities. These initiatives aim to increase awareness and participation in organ donation programs (47). As of 2020, there were 917 patients in Israel awaiting a kidney transplant. There were 173 live donor donations and 257 deceased donor donations (50). Only 10% of the population had signed Adi donor consent cards (51).

## 3 Methods

### 3.1 Study design – measuring economic outcomes

#### 3.1.1 The empirical model – cost–benefit analysis, contingent valuation, and willingness to accept

In this paper, the effects of kidney transplantation on personal well-being are examined through a cost–benefit analysis (CBA). CBA has been a prominent tool in welfare economics for several decades, producing practical outcomes (52, 53). By using CBA, we can gauge an intervention’s advantages in terms of monetary units that match the costs.

To determine these values, two main methods can be used. The first method involves using market information, which is also known as the ‘hedonic’ or ‘revealed preference’ technique. The second method involves conducting an experimental survey, which is referred to as the ‘contingent valuation’ (CV) technique. After considering both methods, we determined that the CV method is the most appropriate for our current study. This is because it is a simple and flexible non-market valuation method that has become the primary technique for monetarily valuing healthcare benefits. This method is particularly useful when dealing with services or goods that are not traded on the market or when legal constraints limit market choices, and the market price does not accurately reflect the value (54–56).

The CV method has faced criticism due to concerns about the accuracy and consistency of its results, as well as the impact of bias and errors. To address these concerns and ensure reliable predictions of kidney transplantation value, we followed the guidelines and recommendations outlined by the US National Oceanographic and Atmospheric Administration (NOAA) panel (57). The panel’s evaluation of the CV method for estimating nonuse values indicated that it can be a valuable tool when used properly, as long as the guidelines are followed carefully throughout the study.

The assessment of a CV is usually based on either WTA (willingness to accept) or WTP (willingness to pay). WTA refers to the minimum amount of compensation a person would require giving up or sell a good or service, while WTP refers to the maximum amount a person would pay to acquire a product or service. In this particular study, a WTA survey was conducted using the payment card technique (PC), which involves presenting respondents with different monetary amounts and asking them to choose their own WTA. This method offers unique advantages because it mimics the purchasing behavior of shopping (58, 59) and allows for value uncertainty. It is especially helpful in the healthcare sector, where individuals may not have prior experience with the intervention being evaluated. Other elicitation methods for CV include the bidding technique, open-ended questions, and dichotomous choice-closed-ended questions. It is important to consider the presence of “range bias” in payment card methods (49, 60), which can influence WTA responses based on the range of presented amounts. To mitigate this risk, the range of presented amounts was determined based on insights from a preliminary round of questionnaires.

Using CBA, it becomes feasible to quantify outcomes in monetary value (49). The net benefit is the monetary difference between the total benefits and total costs. For a healthcare intervention, product, or service to be economically viable, the benefits should surpass the costs, resulting in a positive net benefit. The value of a good to an individual is measured by their minimum WTA for that particular good.

When assessing healthcare interventions that have no private market, alternative methods must be used to establish WTA. In cases where conventional markets for health goods and services do not exist, respondents are asked hypothetical questions about the minimum amount of money they would accept for a good. This approach enables the elicitation of respondents’ values and preferences, as well as the public’s attitude towards various health interventions. Overall, it allows for an evaluation of the perceived health benefits of these interventions (52, 61–66).

In this study, we aimed to gain insight into how people perceive kidney donation. To achieve this, we conducted a survey using the CV payment card (PC) technique. The WTA surveys were conducted in three stages of data collection. The preliminary stage involved identifying the research questionnaire items. This was done through in-depth interviews with five kidney transplant experts, and the questionnaires were designed based on content analysis of the interview results.

#### 3.1.2 Pilot study

We carried out a pilot study in Israel with 38 participants to assess the difficulty and clarity of the research questionnaires, as well as the participants’ willingness to answer them. The goal was to gather detailed information through personal interviews about issues related to kidney transplantation. These interviews, conducted by the researchers, yielded important insights into the research questions and helped determine the credibility and quality of the values obtained from the WTA survey. By conducting face-to-face interviews, we were able to present the participants with information in a controlled way and this enabled us to get responses to complex factors.

#### 3.1.3 Main survey

After conducting a pilot study, we made adjustments to the research questions and developed the final version of the survey. The main survey consisted of questions related to the WTA and demographic factors that may play a role, such as age, gender, education level, income, employment status, ethnicity (Jewish and Muslim), religiosity level, and number of children. All questions on the research questionnaire were presented in a multiple-choice format.

### 3.2 Sampling techniques data collection, and data analysis

#### 3.2.1 For our study, we utilized a combination of snowball and convenience sampling techniques

We posted an advertisement on social media, and those who were interested were asked to complete an online questionnaire. The inclusion criteria for our study were individuals who were 18 years of age or older.

#### 3.2.2 Ethical approval

The participants in the study were given anonymous, self-administered questionnaires, without any intervention. They were given guarantees that no personal or identifying information would be disclosed during data collection and analysis. The cover letter accompanying the questionnaire also informed them that their answers would only be used for scientific research purposes after statistical processing. Participants were given the freedom to choose whether they wanted to participate in the study and provided their written informed consent. The study was approved by the IRB ethic committee, with the ethical approval number 2020026IRB. Out of 1,100 distributed questionnaires, 985 valid questionnaires (89%) were completed by the general public.

#### 3.2.3 Data collection and study questionnaire

We created an online version of the research questionnaire using the Qualtrics software. Links to the survey were distributed on social media, and respondents shared the link with other potential participants and invited them to take part in the research project. Data was collected over a six-month period, from October 2020 to April 2021.

For the chapters in the questionnaire that are relevant to the current paper, please see Appendix 1.

In Appendix 1, we present:

1. The preliminary section to the questionnaire.
2. The Demographic, and Socio-Economic Characteristics in the questionnaire.
3. The Monetary Evaluation of Kidney Donation section in the questionnaire.

#### 3.2.4 Data analyses

The datasets used for compiling the data were obtained from complete surveys only. As a result, there were no missing values in the data files. The statistical analyses in the current study were conducted based on several considerations. A chi-square test is used when in a relationship one of the variables is nominal (e.g., religion) and the other is either nominal, or ordinal (e.g., education level). A binary logistic regression is used when the criterion is binary or dichotomous (i.e., only two response categories) to predict the probability of being allocated to one response group or the other. Regardless, frequencies and descriptive statistics are used to describe the sample and related constructs and variables. The analyses were done in SPSS statistical software (v. 28).

## 4 Results

### 4.1 Demographic information

Several indicators used in the analyses, and their categories are as follows:

#### 4.1.1 Descriptive statistics for sociodemographic information

Gender (0 = male, 1 = female), age, marital status (1 = in a marital relationship of some kind, 2 = not in a marital relationship), number of children, nationality (0 = Jewish, 1 = Arab), religiosity (1 = secular, 2 = traditional, 3 = religious), education (0 = non-academic, 1 = academic), employment status (1 = collecting disability, 2 = pensioner), job tenure, income (1 = 0$–1608.50$, 2 = 1608.81$–3,217$, 3 = 3217.31$–5438.27$, 4 = 5438.58$–6434.01$, 5 = 6434.32$ and above).

**Participants:** There were 985 participants in the study, 50.3% females and 49.7% males between the ages of 18–80 years (M = 42.51, SD = 18.20), while 59.9% are in some type of a marital relationship (e.g., married, couples), and 40.1% are not in any kind of marital relationship (e.g., divorced, single), with number of children between 0–11 (M = 2.41, SD = 2.25). Also, nearly half were Arabs (49.1%), the rest were Jewish; they are either secular (39.4%), traditional (28.4%) or religious (32.2%); nearly half did not have any academic /education or degree (47%); all the other participants did. In terms of work, most of them are employed (73.1%), the rest are unemployed, with job tenure between 1–30 years (M = 8.02, SD = 5.29) with salaries of: (1) $0–$1608.50 (36.8%), (2) $1608.81–$3,217 (32.4%), (3) $3217.31–$5438.27 (11.7%), (4) $5438.58–$6434.01 (14.1%), and (5) $6434.32 and above (5.1%). The data is also depicted in Table 1.

**Table 1:** Sociodemographic information.

Variable	Category	*N*	%	M	SD	*R*
Gender	Female	495	50.3	–	–	–
	Male	490	49.7	–	–	–
Relationship status	Not in a relationship	395	40.1	–	–	–
	In a relationship	590	59.9	–	–	–
Nationality	Jewish	501	50.9	–	–	–
	Arab	484	49.1	–	–	–
Religiosity	Secular	388	39.4	–	–	–
	Traditional	280	28.4	–	–	–
	Religious	317	32.2	–	–	–
Education	Non-academic	463	47.0	–	–	–
	Academic	522	53.0	–	–	–
Employment status	Unemployed	265	26.9	–	–	–
	Employed^1^	720	73.1	–	–	–
Monthly household income^2^	$0–$1608.50	362	36.8	–	–	–
	$1608.81–$3,217	319	32.4	–	–	–
	$3217.31–$5438.27	115	11.7	–	–	–
	$5438.58–$6434.01	139	14.1	–	–	–
	$6434.32 +	50	5.1	–	–	–
Age	–	–	–	42.51	18.20	18–80
Number of children	–	–	–	2.41	2.25	0–11
Job tenure	–	–	–	8.02	5.29	1–30

*N* = frequency; % = relative frequency; M = mean; SD = standard deviation; *R* = range. (1) Employed (working) individuals = full- or part-time or self-employed. (2) https://www.xe.com/ was used for currency exchange rates to $ (USD).

#### 4.1.2 Descriptive statistics for theoretical claims that explain willingness to donate a kidney

The measurement of theoretical claims is carried out using a Likert-type scale ranging from 1 to 7, where 1 denotes no agreement and 7 suggests complete agreement. The scale’s median value is 4, which divides the scores into two categories: low and high. A score below 4 is considered low, while a score above 4 is considered high. For instance, a score of 4.31 on a question related to the importance of people helping each other indicates a high median group, denoting that the respondent considers helping others essential. On the other hand, a score of 2.02 on a question related to the willingness to donate a kidney to anyone in need, regardless of religion, race, or gender, belongs to the low median group, indicating a lack of inclination to donate a kidney to anyone in need. “I see importance in people helping each other” (M = 4.31; SD = 2.73); “I was educated on the values of giving” (M = 6.70; SD = 0.58); “Giving to others gives me satisfaction” (M = 6.70; SD = 0.59); “I personally know people who need a donation” (M = 2.21; SD = 1.61); “Helping someone in need improves my self-esteem” (M = 6.51; SD = 0.81); “The donation gives the opportunity to do something of value” (M = 6.52; SD = 0.82); “I’m willing to donate a kidney only to a first-degree relative (child/sibling/parent)” (M = 5.17; SD = 2.45); “I’m willing to donate a kidney to a second-degree relative (uncle/aunt/cousin/grandparent/ grandchild)” (M = 2.05; SD = 1.67); “I’m willing to donate kidney to a distant relative or friend” (M = 2.23; SD = 2.26); “I’m willing to donate a kidney only to someone I know personally” (M = 4.160; SD = 2.85); “I’m willing to donate a kidney only to someone from my own people” (M = 3.02; SD = 2.83); “I’m willing to donate a kidney to anyone in need, regardless of religion, race, or gender” (M = 2.02; SD = 2.26); “I do not want to be left with only one kidney because it reduces my resilience and impairs my health” (M = 5.31; SD = 2.40); “I do not trust the doctor and the medical team” (M = 1.24; SD = 0.61); “I need time to think and study the kidney transplant’s medical procedure” (M = 5.80; SD = 2.22); “I am not interested in donating a kidney for religious considerations” (M = 1.38; SD = 1.35); “I am not interested in donating a kidney because I am scared of the complications from doing so” (M = 5.29; SD = 2.40); “I am not interested in donating a kidney because of fear of the surgery” (M = 5.28; SD = 2.40); “I am not interested in donating a kidney because I do not want my organ to be implanted into someone else’s body” (M = 1.01; SD = 0.07); “I am not interested in donating a kidney because of objection by my family” (M = 4.930; SD = 2.39). The information is presented also in Table 2.

**Table 2:** Descriptive statistics for theoretical claims that explain willingness to donate a kidney.

Theoretical claims	M	SD
I see importance in people helping each other	4.31	2.73
I was educated on the value of giving	6.70	0.58
Giving to others gives me satisfaction	6.70	0.59
I personally know people who need a donation	2.21	1.61
Helping someone in need improves my self-esteem	6.51	0.81
The donation gives the opportunity to do something of value	6.52	0.82
I’m willing to donate a kidney only to a first-degree relative (child/sibling/parent)	5.17	2.45
I’m willing to donate a kidney to a second-degree relative (uncle/aunt/cousin/grandparent/grandchild)	2.05	1.67
I’m willing to donate kidney to a distant relative or friend	2.23	2.26
I’m willing to donate a kidney only to someone I know personally	4.16	2.85
I’m willing to donate a kidney only to someone from my own people	3.02	2.83
I’m willing to donate a kidney to anyone in need, regardless of religion, race, or gender	2.02	2.26
I do not want to be left with only one kidney because it reduces my resilience and impairs my health	5.31	2.40
I do not trust the doctor and the medical team	1.24	0.61
I need time to think and study the kidney transplant’s medical procedure	5.80	2.22
I am not interested in donating a kidney for religious considerations	1.38	1.35
I am not interested in donating a kidney because I am scared of the complications from doing so	5.29	2.40
I am not interested in donating a kidney because of fear of the surgery	5.28	2.40
I am not interested in donating a kidney because I do not want my organ to be implanted into someone else’s body	1.01	0.07
I am not interested in donating a kidney because of objection by my family	4.93	2.39

All the items are on a 7-level Likert-type scale.

#### 4.1.3 Additional demographic information

Furthermore, the participants were asked three independent questions regarding their willingness to accept payment for a kidney donation. The questions and their respective answers (with relative proportions) are depicted as follows (notably, https://www.xe.com/ was used for currency exchange rates to $ [USD]).

Question #1 (“What is the lowest (minimal) amount that will encourage you to donate one of your kidneys”): (1) Not willing to donate my kidney while I’m still alive (90.9%); willing to donate my kidney while I’m still alive for the amount of (2) $0–$3063.81 (1.2%); (3) $3064.12–$6127.63 (2.4%); (4) $6127.93–$9191.44 (2.6%); (5) $9191.75–$12255.26 (1.4%); (6) $12255.56–$15319.07 (0.6%); (7) $15319.38–$18382.88 (0.8%); (8) $18383.19–$21446.70 (1.2%); (9) $21447.00–$24510.51 (2.4%); (10) $24510.82–$27574.33 (2.6%); and (11) $27574.63–$30638.14 (1.4%).

Question #2 (“What is the lowest (minimal) amount that will encourage you to donate one of your kidneys, after your death”): (1) Not willing to donate my kidney after my death (41.1%); and (2) I am not willing to donate a kidney after my death for payment (58.9%).

Question #3 (“What is the lowest (minimal) amount that will encourage you to donate one of your relatives’ kidneys (child, brother/sister, partner/spouse, father/mother), after their death”): (1) Not willing to donate my relatives’ kidney after their death (41.1%); and (2) I am willing to donate a kidney of a relative after their death without receiving any payment (58.9%).

### 4.2 Logistic regression models

Two (binary) logistic regression models were conceived in order to predict:

1. What is the lowest (minimum) amount that would make you consider donating your kidney *after your death*?
2. What is the lowest (minimum) amount that would make you consider donating a kidney of one of your first-degree relatives (child/brother/spouse/mother/father) *after their death?*

The predictors included: gender, age, marital status, number of children, nationality, religiosity, education, employment status, job tenure, income, and Adi card membership.

Interval-scale items:

Would you be willing to consider an *altruistic* kidney donation (without any payment received?)

“I see importance in people helping each other”; “I was educated on the values of giving”; “Giving to others gives me satisfaction”; “I personally know people who need a donation”; “Helping someone in need improves my self-esteem”; “The donation gives the opportunity to do something of value”; “I’m willing to donate a kidney only to a first-degree relative (child/sibling/parent)”; “I’m willing to donate a kidney to a second-degree relative (uncle/aunt/cousin/grandparent/grandchild)”; “I’m willing to donate kidney to a distant relative or friend”; “I’m willing to donate a kidney only to someone I know personally”; “I’m willing to donate a kidney only to someone from my own people”; “I’m willing to donate a kidney to anyone in need, regardless of religion, race, or gender”; “I do not want to be left with only one kidney because it reduces my resilience and impairs my health.”; “I do not trust the doctor and the medical team”; “I need time to think and study the kidney transplant’s medical procedure”; “I am not interested in donating a kidney for religious considerations”; “I am not interested in donating a kidney because I am scared of the complications from doing so”; “I am not interested in donating a kidney because of fear of the surgery”; “I am not interested in donating a kidney because I do not want my organ to be implanted into someone else’s body”; and “I am not interested in donating a kidney because of objection by my family”.

It must be noted, that since the distributions and statistical information (e.g., frequencies, *p*-values, slopes, standard errors, etc.) are identical for the two questions:

1. What is the lowest (minimum) amount that would make you consider donating your kidney *after your death?* and
2. What is the lowest (minimum) amount that would make you consider donating a kidney of one of your first-degree relatives (child/brother/spouse/mother/father) *after their death*?

Table 3 represents both questions, as the results are identical as well.

**Table 3:** Results of logistic regression model in predicting the lowest (minimal) amount that will encourage donation of one kidney *after death.*

Predictor	*B*	*SE*	Wald	*Sig.*	Exp (*B*)
Gender _(ref = 1st category)_	0.12	0.27	0.18	0.671	1.12
Age	−0.01	0.02	0.72	0.397	0.99
**Marital status**_(ref = 1st)_	**−0.55**	**0.23**	**5.60**	**0.018**	**0.58**
Number of children	0.03	0.08	0.20	0.652	1.03
**Nationality**_(ref = 1st)_	**−3.24**	**1.01**	**10.34**	**0.001**	**0.04**
Religiosity _(ref = 1st)_	−0.27	0.38	0.49	0.486	0.77
Education _(ref = 1st)_	−0.07	0.28	0.06	0.813	0.94
Employment status _(ref = 1st)_	0.23	0.98	0.05	0.819	1.25
Job tenure	0.01	0.03	0.11	0.738	1.01
*Income*_(ref = 1st)_					
$1608.81–$3,217	−0.20	0.30	0.45	0.502	0.82
$3217.31–$5438.27	0.08	0.72	0.01	0.909	1.09
$5438.58–$6434.01	−0.52	0.69	0.56	0.454	0.59
$6434.32 and above	0.30	0.77	0.16	0.693	1.36
**Adi card**_(ref = 1st)_	**4.81**	**1.17**	**16.95**	**0.000**	**22.91**
Would you be willing to consider an *altruistic* kidney donation (without any payment)?	0.03	0.20	0.02	0.897	1.03
I see mutual help as important.	−0.11	0.17	0.42	0.518	0.90
I was educated on the values of giving.	−0.37	0.48	0.61	0.436	0.69
Giving to someone else provides me with satisfaction.	0.42	0.48	0.78	0.376	1.53
**I personally know people who need a donation**	**0.54**	**0.12**	**19.42**	**0.000**	**0.58**
Helping someone in need of aid improves my self-esteem.	−0.43	0.25	2.97	0.085	0.65
The donation gives the opportunity to do something of value.	0.29	0.24	1.48	0.224	1.34
I do not want to be left with one kidney because it diminishes my physical resilience and impairs my health.	0.46	0.31	2.14	0.143	1.58
**I do not trust the doctors and the medical team.**	**−0.54**	**0.16**	**11.00**	**0.001**	**0.58**
I need time to think and study the kidney transplant’s medical procedure.	0.14	0.19	0.60	0.439	1.15
I am not interested in donating a kidney for religious considerations	0.00	0.07	0.00	0.971	1.00
I am not interested in donating a kidney because I am scared of the complications from doing so.	0.03	0.40	0.01	0.943	1.03
I am not interested in donating a kidney because of fear of the surgery.	−0.63	0.43	2.13	0.145	0.53
I am not interested in donating a kidney because I do not want my organ to be implanted into someone else’s body	−0.83	1.20	0.48	0.490	0.44
**I am not interested in donating a kidney because of resistance from my family.**	**−0.40**	**0.11**	**13.51**	**0.000**	**0.67**
*Constant*	11.33	3.66	9.56	0.002	83.25
Model summary: c^2^(29) = 526.19, *p* = 0.000, Nagelkerke’s *R*^2^ = 0.56					

Ref = reference category. 1st = first. Bolded rows indicate a significant effect. Gender (0 = male, 1 = female). Marital status (1 = in a marital relationship of some kind, 2 = not in a marital relationship). Nationality (0 = Jewish, 1 = Arab). Religiosity (1 = secular, 2 = traditional, 3 = religious). Education (0 = non-academic, 1 = academic). Employment status (1 = collecting disability, 2 = pensioner). Income (1 = 0$–1608.50$, 2 = 1608.81$–3,217$, 3 = 3217.31$–5438.27$, 4 = 5438.58$–6434.01$, 5 = 6434.32$ and above). Adi card membership (0 = no, 1 = yes).

Table 3 indicates the following statistically significant effects:

1. *Marital status* (negative relationship): individuals not in a relationship have a *higher* probability to donate their kidney (after death) than those who are currently in a relationship.
2. *Nationality* (negative relationship): Jewish individuals have a *higher* probability to donate their kidney than Arab individuals.
3. *ADI card membership (negative relationship):* individuals without the Adi card have a *higher* probability to donate their kidney than those with the card.
4. *I personally know people who need a donation (positive relationship):* the more people in need of a donation whom the individual knows, the *higher* the probability that they will donate their own kidney.
5. *I do not trust the doctors and the medical team (negative relationship):* the more trust the individual has in the doctors and the medical team, the higher the probability that they will donate their kidney, and vice versa.
6. *I am not interested in donating a kidney because of resistance from my family (negative relationship):* the less resistance from within the family the individual has about donating their kidney, the *higher* the probability that they will donate their kidney.

### 4.3 Supplementary analyses

Additional analyses are provided to elucidate peripheral or otherwise implicit findings. First, frequency analysis revealed that:

1. For the lowest (minimum) amount that would make a person consider donating his kidney? both the median and mode (90.9%) response category is “not willing to donate my kidney while I’m *still alive*.”
2. For the lowest (minimal) amount that would make a person consider donating one of his kidneys, after his death, both the median and mode (58.9%) response category is “I am willing to donate my kidney after my death without receiving any payment.”
3. For the lowest (minimal) amount that would make a person consider donating one of his relatives’ kidneys (child, brother/sister, partner/spouse, father/mother), after their death, both the median and mode (58.9%) response category is “I am willing to donate a kidney of a relative after their death without receiving any payment.”

Moreover, in order to evaluate the connection between income level and the lowest (minimal) amount that would make a person consider donating a kidney, a chi-square test was calculated.

The results indicate a significant association between the two factors: c^2^ (24, *N* = 985) = 95.97, *p* = 0.000, *r_c_* = 0.16. Meaning, those with an income between $0–$1608.50 preferred to receive monetary compensation for a kidney donation, those between $1608.81–$3,217 and between $3217.31–$5438.27 preferred **not** to receive such compensation, those between $5438.58–$6434.01 and $6434.32+ were indifferent.

In order to evaluate the connection between income level and the lowest (minimal) amount that would make a person consider donating his kidney after his death, a chi-square test was calculated. The results indicate a significant association between the two factors: c^2^ (4, *N* = 985) = 104.33, *p* = 0.000, *r_c_* = 0.33. Meaning, those who were unwilling to have a kidney donated after their death are from income levels of $0–$1608.50 and $5438.58–$6434.01 while the rest (i.e., $1608.81–$3,217, $3217.31–$5438.27 and $6434.32) were willing to donate their kidney after death.

In order to assess the link between income level and the lowest (minimal) amount that will encourage a person to donate one of his relatives’ kidneys (child, brother/sister, partner/spouse, father/mother), after their death, a chi-square test was calculated. The results indicate a significant association between the two factors: c^2^ (4, *N* = 985) = 104.32, *p* = 0.000, *r_c_* = 0.32. Meaning, those who were not willing to donate a kidney after their death are from income levels of $0–$1608.50 and $5438.58–$6434.01, while the rest (i.e., $1608.81$–$3,217, $3217.31–$5438.27 and $6434.32+) were willing to have their kidney donated after their death.

In order to evaluate the connection between income level and religion, a chi-square test was calculated. The results indicate a significant association between the two factors: c2 (4, *N* = 985) = 217.56, *p* = 0.000, rc = 0.47. which shows that, Muslims have less income, on average, than Jews: Muslims are at a $0–$1608.50 income level, while Jews are at the other levels (1608.81$–$3,217, $3217.31–$5438.27, 5438.58–$6434.01 and $6434.32+).

In order to evaluate the connection between income level and religiosity, a chi-square test was calculated. The results indicate a significant association between the two factors: c2 (8, *N* = 985) = 462.50, *p* = 0.000, rc = 0.49. This indicates that religious individuals earn lower salaries than secular individuals, who earn lower salaries compared to traditional (religiously) people. Religious people tend to have income levels of 0–$1608.50, more so than secular individuals. Secular people are at income levels of 0–$1608.50, $1608.81–$3,217, $3217.31–$5438.27, while traditional individuals are more likely to have income levels ranging from $3217.31–$5438.27, $5438.58–$6434.01 to $6434.32 + .

In order to evaluate the connection between income level and education levels, a chi-square test was calculated. The results indicate a significant association between the two factors: c2 (12, *N* = 985) = 441.17, *p* = 0.000, rc = 0.39. In other words, an increase in education level is often followed by an increase in salary (e.g., academically educated individuals are salaried more than non-academically educated people): those who are not academic are on 0–$1608.50 and 1608.81$–$3,217 income levels, and academically educated individuals are on 3217.31–$5438.27, 5438.58–$6434.01 and $6434.32 + .

**Figure 1:**
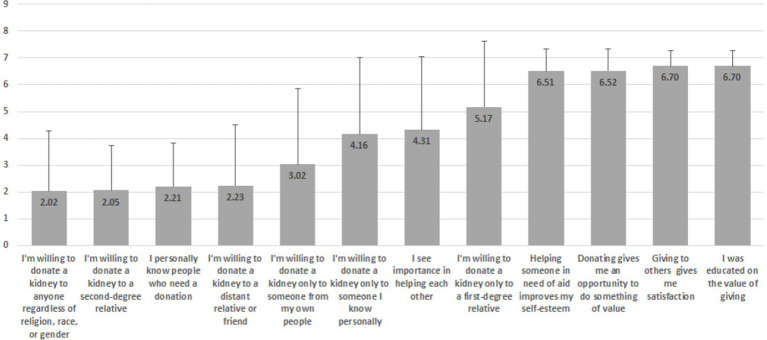
Ranking of responses from least to most important.

**Figure 2:**
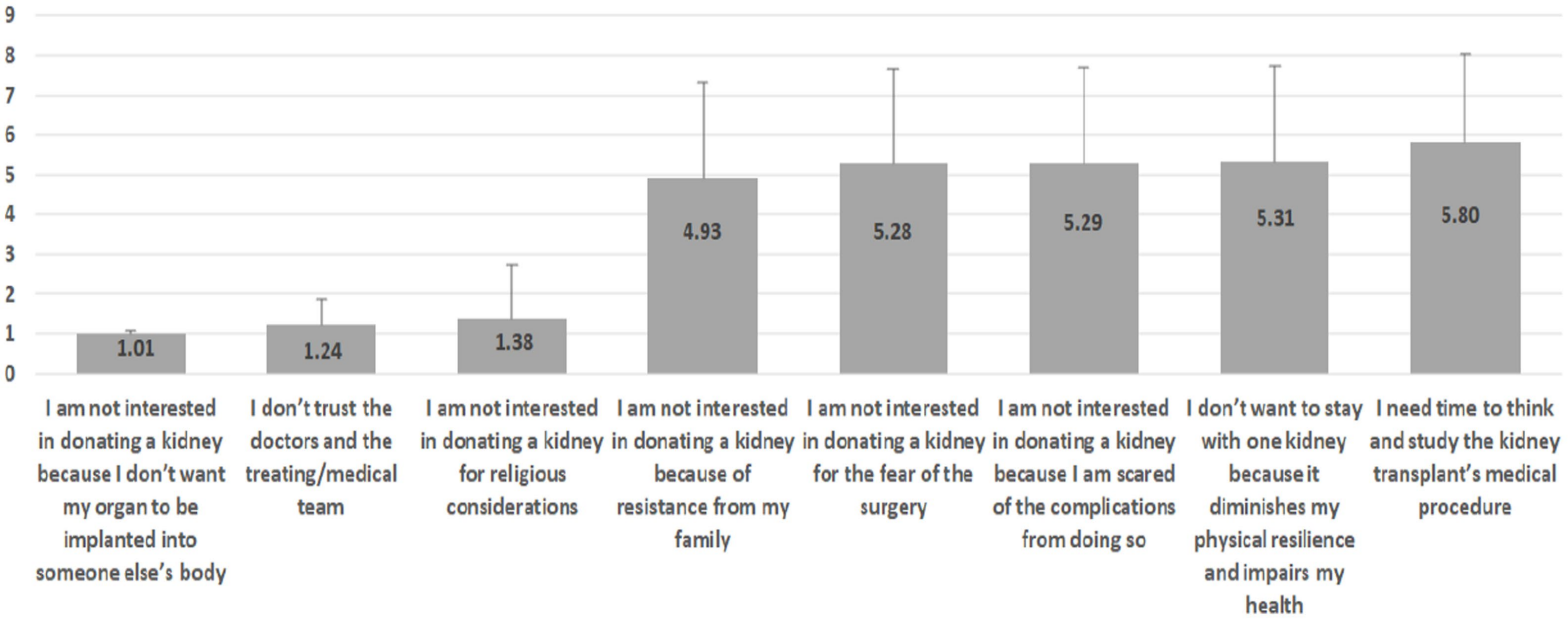
Ranking of responses from least to most important.

Additionally, since the majority were not willing to donate a kidney while they were still alive, two sets of rankings were made, as illustrated in Figures 1, 2. The scales of the items depicted in the graphs are Likert-type ranging between 1 (not at all) and 7 (completely agree 100%).

As can be seen in Figure 1, the least important factors are: “I’m willing to donate a kidney to anyone in need, regardless of religion, race, or gender” (M = 2.02, SD = 2.26) and “I’m willing to donate a kidney only to a first-degree relative (child/sibling/parent)” (M = 2.05, SD = 1.67), while the most important are: “Giving to others gives me satisfaction” (M = 6.70, SD = 0.59) and “I was educated on the value of giving” (M = 6.71, SD = 0.58).

On the other hand, in Figure 2, the least important is “I am not interested in donating a kidney because I do not want my organ to be implanted into someone else’s body” (M = 1.01, SD = 0.07) and “I do not trust the doctor and the medical team” (M = 1.24, SD = 0.61), while the most important are “I do not want to be left with only one kidney because it reduces my resilience and impairs my health” (M = 5.31, SD = 2.40) and “I need time to think and study the kidney transplant’s medical procedure” (M = 5.80, SD = 2.22).

Interestingly, in Figure 1 it is noticeable that in the lower rankings there is more consensus (i.e., less heterogeneity) in responses, as reflected by the lower standard deviations, while in the upper rankings it is the opposite. In contradistinction, in Figure 2, more consensus is attributed to the lower rankings as opposed to the upper ones.

Furthermore, based on the indicators in Figures 1, 2, additional zero-order Pearson correlations were calculated, per item group. Results indicate two interesting findings:

1. While item “I’m willing to donate a kidney only to a first-degree relative (child/sibling/parent)” is positively correlated with item “I’m willing to donate a kidney to a second-degree relative (uncle/aunt/cousin/grandparent/grandchild)” (*r* = 0.43, *p* = 0.000), both of them are negatively associated with the item “I am not interested in donating a kidney because of objection by my family”: *r* = −0.71, *p* = 0.000 (notably, a strong relationship) and *r* = −0.25, *p* = 0.000, respectively.
2. While item “I’m willing to donate a kidney only to someone I know personally” is positively (and strongly) correlated with item “I’m willing to donate a kidney only to someone from my own people” (*r* = 0.71, *p* = 0.000), both of them are negatively associated with the item “I’m willing to donate a kidney to anyone in need, regardless of religion, race, or gender”: *r* = −0.50, *p* = 0.000 and *r* = −0.32, *p* = 0.000, respectively.

Lastly, an additional binary logistic regression analysis was employed in order to predict the variables that encourage respondents to donate a kidney.

The predictors included:

“I see importance in people helping each other”; “I was educated on the value of giving”; “Giving to others gives me satisfaction”; “I personally know people who need a donation”; “Helping someone in need improves my self-esteem”; “The donation gives the opportunity to do something of value”; “I’m willing to donate a kidney only to a first-degree relative (child/sibling/parent)”; “I’m willing to donate a kidney to a second-degree relative (uncle/aunt/cousin/grandparent/grandchild)”; “I’m willing to donate kidney to a distant relative or friend”; “I’m willing to donate a kidney only to someone I know personally”; “I’m willing to donate a kidney only to someone from my own people”; “I do not want to be left with only one kidney because it reduces my resilience and impairs my health”; “I do not trust the doctor and the medical team”;

“I need time to think and study the kidney transplant’s medical procedure”; “I am not interested in donating a kidney because I am scared of the complications from doing so”; “I am not interested in donating a kidney because of fear of the surgery”; “I am not interested in donating a kidney because I do not want my organ to be implanted into someone else’s body”; “I am not interested in donating a kidney because of objection by my family.”

The method used was conditional-forward, and, as such, not all predictors are portrayed in the final model. The results are displayed in Table 4.

**Table 4:** Results of logistic regression model that predicts the variables that encourage respondents to donate a kidney, after death.

Predictor	*B*	*SE*	Wald	*Sig.*	Exp (*B*)
I’m willing to donate a kidney only to someone from my own people	0.43	0.03	171.41	0.00	1.54
I do not trust the doctor and the medical team	−0.39	0.13	8.87	0.00	0.68
I am not interested in donating a kidney for religious considerations	−0.13	0.06	5.00	0.03	0.88
I am not interested in donating a kidney because of fear of the surgery	−0.53	0.10	30.12	0.00	0.59
I am not interested in donating a kidney because of objection by my family	−0.42	0.08	28.53	0.00	0.66
*Constant*	5.25	0.59	80.10	0.00	191.24
Model summary: c^2^(7) = 452.88, *p* = 0.000, Nagelkerke’s *R*^2^ = 0.50					

All predictors are continuous on a 7-level Likert-type scale. The regression method used was conditional-forward, which is why all the relevant associations are statistically significant in the table.

Table 4 indicates the following statistically significant effects:

1. **I’m willing to donate a kidney only to someone from my own people (positive relationship):** higher willingness to donate based on a national rationale increases the probability to donate a kidney.
2. **I do not trust the doctor and the medical team (negative relationship):** less trust in the medical team and physicians decreases the probability of a kidney donation.
3. **I am not interested in donating a kidney for religious considerations (negative relationship):** less propensity to donate based on religious traditions decreases the probability of a kidney donation.
4. **I am not interested in donating a kidney because of fear of the surgery (negative relationship):** less propensity to donate based on fear of the surgery decreases the probability of a kidney donation.
5. **I am not interested in donating a kidney because of objection by my family (negative relationship):** less propensity to donate based on resistance from the family decreases the probability of a kidney donation.

## 5 Discussion

In this study, we assessed the public benefits of kidney donation in Israel in order to understand the economic forces at work and to investigate the WTA for kidney donation. The study findings and conclusions, using Israel as a case study, may also be relevant to organ donation in other countries. They reflect religious beliefs and ethical worldviews as well as ethical and legal considerations. This suggests that an examination and explanation of the organ markets should be carried out with respect to these factors. According to WTA theory, the amount of money that a person is willing to accept for providing a product, service, medical procedure or health intervention is a yardstick of what value the general public assigns to that that benefit (57). The US National Oceanographic and Atmospheric Administration (NOAA) panel found that WTA decisions are affected by a variety of motivations, among them ethical and moral concerns. The consumers’ willingness to accept is often affected by moral considerations (67). Social and personal norms similarly have considerable influence on the WTA (68). Contingent valuation responses indicate the willingness to accept for the moral satisfaction of contributing to the public welfare (69).

Policy makers must justify the costs versus benefits of interventions before authorizing them (70). so that assessing the WTA for kidney donation is imperative for health policy makers in their deliberations over state funding for kidney transplantation out of the limited national medical budget. Our results support other research (71) that argue that most respondents replied that they were not willing to donate an organ for money during their lifetime. Thus, the present study the respondents’ lack of support for commercializing organ donation. Accepting a substantial payment for providing a kidney was regarded as intrinsically wrong because it seems to denote turning the human body into another commodity and a diminution of human dignity (1).

### 5.1 Objections to accepting payment for kidney donation

#### 5.1.1 Ethical objection

The study participants did not provide a theoretical explanation for their objections to payment. However, objections to the commodification of body parts are grounded in Kant’s second categorical imperative relates to the concept of objectification or of treating others as a means, Objectification breaches the moral principle of respect for human beings, seeing the human being as an object as a means to an end rather than possessing a value in their own right, and then disrespects human dignity by putting a price on it (72). Transactions in human body parts for money violates human dignity because people are treated as abstract resources, without subjectivity, autonomy, and agency. There is a viewpoint that opposes financial compensation for organ donors, particularly for their kidneys. This is because body parts are closely linked to individual human being, who have inherent worth and dignity. The act of selling body parts reduces individuals to the status of commodities, which is incompatible with their inherent worth and dignity.

Although the participants did not explicitly state that donated kidneys could not be transferred, they shared their feelings of being degraded and disrespected in various ways. Several expressed sorrow over losing their sense of personal pride because of their inability to care for themselves and their loved ones after the procedure, as they lacked the financial means to do so. This often resulted in them seeking external aid, which could compromise their decision-making independence when it came to donating their kidneys. Hence, portraying organ donation as an act of selflessness while disregarding financial compensation may have unintended consequences on donors’ social and financial welfare, along with their emotional well-being and sense of value.

#### 5.1.2 Objection to violation of equity

The findings of our research contradict the market approach, which prioritizes availability of organs to those who can afford it through personal funds or private insurance. This approach heavily emphasizes individual rights, while minimizing the importance of equity and fairness. Our research demonstrates several reasons for rejecting the market approach. Investments from public taxes have been made in transplant technologies during their research and development. Yet, if these medical resources are solely utilized for transplantation, it may result in a shortage of resources for other urgent medical treatments. It must be stated that having sufficient financial means should not be the sole basis for applying for a kidney transplant. However, the most concerning issue with this approach is that it ignores the values of fairness and equity. It is degrading for patients who are lacking financial means to have to rely on public campaigns to raise the money for their transplant. This not only diminishes the patients’ dignity but also reflects poorly on the society that allows it. Making financial ability the sole determinant of access to life-saving treatments implies that society has put a price tag on human life, which is unacceptable. This is particularly true in a society where there are large disparities in income (73).

### 5.2 Arguments favoring incentives for donation

#### 5.2.1 Balancing altruism and personal interest

An argument in favor of equal access to organs for transplantation is the notion that organs are donated for the benefit of the public. The call to action is for individuals, regardless of their financial status, to donate their organs. This perspective considers organs as a public resource that should be equally accessible to all, including those who cannot afford the medical procedure (48).

It is worth noting that only a small percentage (18.5%) of the general public is willing to donate a kidney for payment, according to recent research. This finding is particularly interesting in light of Israel’s policy of incentivizing donors. It seems that Israel recognizes the complex motivations behind organ donation and seeks to reward those who choose to give in this way (74).

The act of donation involves a balance between altruistic intent and personal interest. Due to its significant impact on patients and society, the shortage of organs is recognized as a critical public health challenge (29, 75).

The regression model yielded interesting results as to the significant predictors of participants’ responses to two questions: (1) What is the lowest (minimal) amount that would encourage you to donate a kidney after your death: and (2) What is the lowest (minimal) amount that would encourage you to donate a kidney of a deceased family member (child, brother/sister, partner/spouse, father/mother) after their death. The answer lies with nationality – the Jewish respondents expressed a higher likelihood of donating a kidney than Arab respondents.

### 5.3 Religious considerations

#### 5.3.1 Altruism

Nationality plays a strong role in formation of concepts of identity and belonging, which, in turn, impact the individual’s approach to the concept of donating an organ (76, 77). The formal definition used here for “religion” is having belief in a Divinity and a commitment to one of the organized religions (78). Israel is home to two major religions, Judaism, and Islam, which both ascribe the highest sanctity to human life and to the value of saving a life. Consequently, the medical definition of brain death has gained increased acceptance among Muslim religious scholars who permit organ donation from deceased donors (79). Jewish religious authorities likewise sanction organ donation among their believers.

#### 5.3.2 Religious restrictions and contraindications on organ donation

Yet both religions place restrictions which are intended to proscribe the desecration of the human body, either living or posthumously. These limitations prompt reluctance among religiously observant people, both Muslim and Jewish, to donate organs for fear that this will lead to such desecration (80, 81). For example, in certain groups of ultra-Orthodox Jews, defining the exact moment of death remains a matter of dispute, whether it is brain death or cardiorespiratory death that determines the end of life. Some traditional Jewish views hold that taking the organs of a deceased person is a desecration of the divine image of God in man (82). Similarly, a significant number of Muslims believe that the Islamic sharia forbids organ donation, because there is no mention of it in the Qur’an. Moreover, a tenet of Islamic faith is that only God decides the fate of a dead body. Religious Muslims attribute supreme importance to the “intactness of the body in the afterlife” (83). Muslims furthermore explain their unwillingness to consent to organ donation by a religious belief that the dead will be resurrected and thus the physical body must be preserved intact. Many also believe that the sick are healed only by the will of God. Finally, the dead should be buried immediately, and organ donation delays the funeral, a further desecration of the dead (84).

The findings of the present study show that Jewish respondents show more willingness to donate a kidney than their Arab counterparts. This suggests that Jewish respondents had more positive attitudes toward kidney donation than Muslims. These findings align with previous studies that showed differences in the attitudes to organ donation by different national religious groups.

#### 5.3.3 Living kidney donation and nationalism

In an ideal world, all organ donations would be unconditional and allocated based solely on the recipient’s need (85). However, in reality, the majority of organ donations, both from living and deceased donors, are conditional. The allocation system often sets its own conditions, such as prioritizing local patients or those willing to donate to the organ pool over “free riders” (45). Conditional organ donation can be directed to an individual with specific terms and conditions or to specific groups or people. This latter form, called “sectarian donation” is almost always in breach of the prevailing international transplant ethic. While some argue that divisive donations still save lives (86–88), the current international transplant ethic codes consider this immoral and unacceptable, regardless of the donor’s conditions. Economic considerations, social standing, class, race, ethnicity, faith, gender, nationality, age, reciprocity, friendship, and even kinship would play no role in the decision-making process regarding organ donation (85).

### 5.4 Predictors of willingness to donate an organ

#### 5.4.1 Marital status

Individuals not in a relationship have a higher probability to donate their kidney (after death) than those that are currently in a relationship. Marital status has an inverse relationship to a person’s willingness to donate a kidney. People who are not married are most likely to be willing to donate. In the matrimonial system, the decisions regarding organ donation even after death are more complex since the spouse in the nuclear family and the joint families of the spouses must be considered. In the professional literature, there is a foundation for this understanding (88, 89).

#### 5.4.2 Confidence and faith in the medical team

Limited public awareness has been identified as a contributing factor to the fear and apprehension surrounding living kidney donation. Specifically, the potential donors’ concerns regarding the surgical risks and the long-term health and lifestyle effects of the donation process have been identified as key factors that may lead to hesitation. The general public supports living kidney donation as long as medical professionals facilitate informed decision-making, recognize and address coercion, and conduct objective donor assessments. Physicians must ensure that donors can make informed decisions about the potential risks involved. They should also identify significant medical risks and minimize possible complications and harms (90).

#### 5.4.3 Adi card signing

Individuals without the Adi card have a higher probability to donate their kidney than those with the card. One might explain these findings which seem incongruous, by the complex and conflicted nature of prosocial behavior (91–93). Prosocial behavior may seem to be clear-cut and uncomplicated. One party needs help, another party can give that help it, and when that happens, the helper is demonstrating their character, altruism, and good intentions. However, recent studies have revealed some of the reasons why people turn away from prosocial behavior, and these indicate that self-interested concerns can sometimes outweigh prosocial tendencies. There were times participants did not want to behave as was expected of them when they could not justify it in terms of their self-interest (94). In other cases, the retreat from prosocial behavior may be expressed as an act of self-defense against threats (95). Twenge and colleagues found that certain forms of social exclusion considerably decreased prosocial behavior in many different areas. This effect was affected by empathy but not by the prevailing mood, suggesting that when the individual feels personally threatened, empathy for the plight of others is severely impaired. One categorical example of the ambivalent nature of prosocial behavior is the disinclination to donate organs posthumously. Studies and opinion surveys have regularly showed an odd incongruity between the generally favorable attitude people have toward organ donations and their understanding of their significance, even agreeing to sign an Adi card, in the face of their reluctance to donate organs themselves (96–98). So, given the above, it is possible to explain the findings of our research, which show a negative relationship between signing an Adi card, which is the socially correct act, and a genuine willingness to donate a kidney, even after death.

#### 5.4.4 The relationship between mortality salience and organ donation attitude and behavior

Hirschberger and colleagues (91, 98, 99) hypothesize that the effect of mortality salience, or awareness of the inevitability of death, on prosocial behavior is contingent on what this prosocial behavior involves. Thus, if the behavior, such as ethical and altruistic conduct, is helpful in managing the terror of death, mortality salience will increase the behavior, However, if the behavior undermines management of this terror, mortality salience will lessen the prosocial behavior. Hirschberger et al. further explain that organ donation increases death awareness, so confronting the question of whether to donate organs may further exacerbate death anxiety and the consequently, the answer will be refusal, even if they sign a donor card.

#### 5.4.5 Knowing a donor or recipient

the more people, in need of a donation whom the individual knows personally, the higher the probability that they will donate their own kidney. Another predictor of a favorable attitude toward organ donation is having some prior personal experience with it, or knowing someone who has been a recipient or donor (100). Studies (101–105) showed that personal acquaintance with an organ recipient, organ donor, someone either on the transplant waiting list, or who is willing to donate an organ were all predictors of willingness to donate and donor behavior.

#### 5.4.6 Family influence

the less resistance from within the family the individual has about donating their kidney, the higher the probability that they will donate a kidney. The influence of the family’s opinion is of high relevance. “Favorable opinion toward the donation of the father, mother, and partner,” “Having a family member with a donor card,” “Being in favor of donating the organs of a relative,” and “Consulting with family” were cited as significantly related factors. Opinions of family members affected the intentions of subjects on whether they would donate organs (106, 107). Studies (104, 108–110) showed the views of family members and significant others impacted individuals’ attitudes toward organ donation. One predictor of willingness to donate was perceived positive attitudes of a partner toward donation or the belief that a partner that a significant other would support or favor one’s decision to donate an organ. Having a partner advocates organ donation is also a predictor of more positive attitudes. as well as having discussed the subject with family members.

#### 5.4.7 Income

In contrast to research that found no statistical interactions between payment and income (6), an examination of the interaction between income and the willingness to accept payment for kidney donation yields interesting findings. While the majority of the sample population expressed unwillingness to donate a kidney for monetary compensation [these finding has been reported in other academic papers, see (111)], there exists a small percentage of individuals whose income ranges between $0–$1608.50, who preferred to receive a monetary compensation for a kidney donation. Those between $1608.81–$3,217 and between $3217.31–$5438.27 preferred **not** to receive such compensation, those between $5438.58–$6434.01 and $6434.32+ were indifferent. The academic literature indirectly supports the findings. It suggests that individuals with moderate income levels are more likely to oppose kidney donation compared to those with lower incomes (112, 113).

Regarding donating a kidney after death those who were unwilling to have a kidney donated after their death are from income levels of $0–$1608.50 and $5438.58–$6434.01 while the rest (i.e., $1608.81–$3,217, $3217.31–$5438.27 and $6434.32+) were willing to donate their kidney after death.

#### 5.4.8 Sociodemographic factors as predictors of kidney donation

Education and socioeconomic status are significantly associated with organ donation attitudes. There is a wide gap in donation rates based on these factors (114). In our study, we found that prioritization of attitudes towards incentives may differ based on ethnicity/race, in accordance with the income levels of the respondents (113). Our research indicates that individuals with higher academic qualifications tend to earn higher salaries in comparison to those with only a high school education. This implies that people with advanced educational levels typically have a more positive attitude towards organ donation. This conclusion is supported by relevant literature in the field (115). According to our findings, Muslims have a lower average income than Jews. Therefore, we can deduce that Jews are more likely to have a favorable outlook on organ donation. Studies have shown that minorities have significantly lower rates of donation (116, 117). We also discovered that religious individuals earn less than secular individuals, who earn less than traditional (religious) people. This suggests that religion may have an impact on organ donation in both positive and negative ways, as some studies have found that religion can be a factor in favor of organ donation (118, 119) while others have found the opposite (108, 118, 120).

#### 5.4.9 Moral particularism

Most of the respondents are not willing to donate a kidney to any person, regardless of religion, race, and gender. There is a clear preference among respondents to donate a kidney to first- or second-degree family members as well as to people they know and members of their own people. This finding is supported by the literature, which emphasizes that close relatives take precedence over others in organ donation [i.e., (121–123)]. “Moral particularism” refers to the natural disposition prioritize people with whom we have a connection, whether by social group, community, or other type of network (124, 125).

## 6 Conclusion

Recently, licensing authorities have expressed interest in using the cost–benefit analysis (CBA) and willingness-to-accept (WTA) methodologies to evaluate innovative medical procedures. Our research provides strong evidence supporting the reliability and validity of these approaches in assessing the benefits of medical procedures. This is particularly relevant in the context of discussions around funding or subsidizing procedures that are already supported by the healthcare system, given the limited resources available for healthcare services.

Respondents in our survey generally value the concept of helping others and derive satisfaction from giving. Their However, their willingness to donate a kidney varies, and depending depends on their relationship with the potential recipient. There is a greater willingness to give to close family members. Other factors that affect their decision to donate include their trust in medical professionals, health concerns, and fears related to the surgery. Religious and family objections have a relatively lesser influence.

The research indicates that the vast majority of the general population is unwilling to donate a kidney while alive. However, more than half of the population are willing to donate after their death without receiving any payment. This suggests that there is no support among potential donors for the commercialization of organs. The concept of compensating donors is considered ethically problematic due to concerns about commodification of the human body and the potential for the sale of body parts to undermine human dignity.

The implications of the public’s lack of support for organ commercialization and compensation are significant. It means that alternative solutions to address the shortage of organs for transplantation must be explored. This could include initiatives to increase awareness and education around organ donation, as well as efforts to improve the efficiency of the organ procurement system. Additionally, there may be ethical considerations for policymakers to consider when deciding whether or not to legalize organ compensation.

As kidney donation involves complex social, economic, and ethical aspects, we recommend policymakers to continue full funding.

Healthcare professionals and policymakers should consider the impact of various factors on the likelihood of kidney donation after death when designing effective strategies to improve the availability of this life-saving treatment. These factors include a strong sense of national identity, religious considerations, marital status, nationality, membership in the ADI card program, personal connections to potential recipients, trust in medical professionals, and resistance from family members. Understanding these factors can help inform targeted interventions to increase the number of kidney donations, reduce organ shortages, and ultimately improve health outcomes for patients in need of this critical treatment.

Furthermore, income level was found to be significantly associated with the willingness to donate a kidney, with people from different income levels having varying preferences and motivations regarding kidney donation. Policymakers and healthcare professionals should consider these findings when designing strategies and programs to increase kidney donation rates and reduce disparities in access to transplantation.

In conclusion, this study provides valuable insights into the factors that influence the willingness of individuals to donate their kidneys, both in life and after death. The findings suggest that various social, cultural, and economic factors impact an individual’s decision to donate a kidney, and healthcare professionals and policymakers must take these factors into account when developing strategies to increase kidney donation rates. The study also sheds light on the ethical and moral concerns surrounding the commercialization of organ donation, highlighting the need for continued funding of kidney transplantations. Overall, this research contributes to the growing body of literature on organ donation and can be useful for academics, healthcare professionals, and policymakers seeking to improve the availability of life-saving kidney transplantation.

### 6.1 Recommendations for practice

We recommend a policy of continuing full funding for kidney transplantation. It is crucial for healthcare policy makers and dialysis specialist to have a better understanding about the public’s attitudes toward organ donation. To improve these attitudes, a culturally diverse approach must be adopted. Proactive steps should involve education for all the relevant religious and ethnic groups explaining how their values and religious belief are promoted by organ donation (126). Health care organizations and hospitals must offer patients and families the possibility of consulting with religious authorities who are available and competent to guide and supervise procedures when a decision about organ donation must be made.

Consulting with family is seen to influence attitudes on organ donation. Although family members might have a negative attitude, engaging in discussions while the patient is alive to eliminate differences in opinions can result in increased opportunities for organ donation (106, 107). Explicit discussion about donation intentions among family members will have a positive impact on donation rates (127–131). Presumably, appropriate public exposure would result in more family discussion and more frequent declaration of one’s wishes to donate, decreasing uncertainty at the critical time (brain death of a loved one) and likely increasing organ donation (132).

Our analysis regarding the Adi card indicates that although prosocial behavior is highly valued in most, if not all, societies, self-protective concerns may at times override the general positive feeling one has toward helping others in need and lead to defensive withdrawal. Signing an Adi card is a prosocial act and yet, at the moment of truth, personal interest and fear of injury or death led to people deciding not to donate a kidney.

The less distrust the individual has towards the doctors and the medical team, the higher the probability that they will donate their own kidney. It is crucial to comprehend the issue of medical mistrust. Instead of tackling it head-on, it might be more successful to clarify the different protocols in place within the medical and organ allocation system. These measures ensure that doctors cannot harm patients for their organs or engage in illegal organ trading. The organ allocation system also prevents favoritism towards wealthy or famous individuals and discrimination based on race (133). Interventions to improve living organ donation should target potential donors’ concerns about undergoing the surgical procedure and mistrust of and discrimination within hospitals (134).

Deciding whether to donate one’s organs is a personal decision that involves various factors, including religious and cultural beliefs. This can be further complicated by issues such as a lack of trust in the medical system, misunderstandings about religious stances, and a lack of knowledge about the donation process. It’s important to engage with the community, including disadvantaged and minority groups, to build trust and provide information, which can help promote organ donation in the future (135, 136).

## 7 Limitations

- The survey participants filled in a questionnaire online. This effectively limited participation to only patients who were technologically savvy and had internet access.
- The patients who had a greater interest in a market in kidneys were more likely to have participated in a survey related to paying for a kidney than participants without a similar interest.
- Because the questionnaires were relatively long and complex, the number of respondents who completed them may have been affected, although it is still relatively quite high.

## Data availability statement

The raw data supporting the conclusions of this article will be made available by the authors, without undue reservation.

## Ethics statement

The studies involving humans were approved by IRB ethics committee, with the ethical approval number 2020026IRB. The studies were conducted in accordance with the local legislation and institutional requirements. The participants provided their written informed consent to participate in this study.

## Author contributions

LG: Conceptualization, Formal analysis, Investigation, Methodology, Project administration, Supervision, Validation, Visualization, Writing – original draft, Writing – review & editing. YB-C: Conceptualization, Data curation, Resources, Validation, Writing – review & editing. MT: Conceptualization, Data curation, Resources, Validation, Writing – review & editing.
