# Supplementary material for: The general public’s attitude towards accepting payment for kidney donation
Source: Front Med (Lausanne). 2023 Dec 13;10:1282065. doi: 10.3389/fmed.2023.1282065 (PMC10756681; doi:10.3389/fmed.2023.1282065)
Supplement: Supplementary file 1 [file Data_Sheet_1.docx]

Dear respondent,

I am doing research on the economic aspects of kidney transplantation.

The study deals with the valuation of kidney transplantation, while considering the treatment procedure and estimating the minimum amount a donor would be willing to accept for donating a kidney.

The way to put a price on this is an economic estimation method called Willingness to Accept (WTA), which is a possible way to measure benefit that is derived from donating a kidney. This method is based on survey questions, both direct and hypothetical, about the minimum amount the subjects in the sample are willing to accept for kidney donation.

In order to collect information on the kidney transplantation process, I would like to conduct a survey, and I invite you to take part in it.

Your participation in the study is very important.

This survey will be used to raise the issue of your preferences, your needs, and your demands, and it will increase your chances of influencing the quality and availability of the service provided.

The success of the study depends on your cooperation.

I thank you very much for the time you are willing to devote to this study.

It is important for you to know:

The questionnaire does not ask for your name or any other identifying factor.

The questionnaire is completely confidential and will only be used for this study.

I will not test knowledge or skill; I am interested in your thoughts, feelings, and opinions.

There are no correct and incorrect answers. Your personal response is important to me.

The questionnaire is addressed to both men and women.

Thank you again for participating in the study.

1. **Demographic, Socio-Economic and Health Characteristics**

INSTRUCTIONS: This questionnaire gathers basic information about you. Please circle your answer.

1. **Are you**

- Male
- Female

1. **How old are you?** _____
2. **What is your family status?**

- In a couple relationship of some kind
- Not in a couple relationship

1. **Ethnicity**

- Jewish
- Arab

1. **Your degree of religious observance^^[[1]](#footnote-1)^^-** Israelis tend to define their religious affiliation by degree of their religious practice.

- Traditional^[[2]](#footnote-2)^
- Religious^^[[3]](#footnote-3)^^
- Secular^^[[4]](#footnote-4)^^ - Not religiously observant

1. **Number of children** (please specify ages) ____________
2. **What is the highest level of education that you have completed**?

- Non-Academic - Elementary School (1^st^ grade – 9^th^ grade, age range 6-15) and High school graduate (10^th^ grade – 12^th^ grade, age range 16-18)
- Academic - College degree (college, university)

1. **Which of the following best describes your employment status?**

- Employed
- Unemployed

1. **How long have you been working?** ____________
2. **What is your monthly household income?**

- $0–$1608.50
- $1608.81–$3217
- $3217.31–$5438.27
- $5438.58–$6434.01
- $6434.32 +

# **Part Two**

1. Have you signed an ADI organ donor card?

1. Yes

2. No

2. In general, do you think that donating a kidney is mainly a positive or a negative phenomenon?

1. Mainly positive

2. Mainly negative

3. Sometimes positive and sometimes negative

3. Would you be willing to consider donating a kidney **altruistically** (in other words, without being paid)? Only direct expenses would be paid, e.g., travel costs – such as has been defined by law).

| Description | 7  Definitely  100% | 6  To a very great extent | 5  To a great extent | 4  To a moderate extent | 3  To a  small  extent | 2  To a very  small  extent | 1  Not at all |
| --- | --- | --- | --- | --- | --- | --- | --- |

Below is a list of possible reasons for donating a kidney from a living or dead donor without receiving payment (altruistic donation). Please indicate for each one the extent of your agreement with each reason. Remember: there are no 'right' or 'wrong' answers; the answers express different opinions.

4. Circle the extent to which you agree with the statements in the table.

The extent of agreement is rank in ascending order from 1 to 7.

| Description | 7  Definitely  100% | 6  To a very great extent | 5  To a great extent | 4  To a moderate extent | 3  To a  small  extent | 2  To a very  small  extent | 1  Not at all |
| --- | --- | --- | --- | --- | --- | --- | --- |
| 1. I see importance in people helping each other. | 1 | 2 | 3 | 4 | 5 | 6 | 7 |
| 2. I was educated on the value of giving. | 1 | 2 | 3 | 4 | 5 | 6 | 7 |
| 3.Giving to others gives me satisfaction. | 1 | 2 | 3 | 4 | 5 | 6 | 7 |
| 4. I personally know people who need a donation. | 1 | 2 | 3 | 4 | 5 | 6 | 7 |
| 5.Helping someone in need improves my self-esteem. | 1 | 2 | 3 | 4 | 5 | 6 | 7 |
| 6. The donation gives the opportunity to do something of value. | 1 | 2 | 3 | 4 | 5 | 6 | 7 |
| 7.I'm willing to donate a kidney only to a first-degree relative (child/sibling/parent) | 1 | 2 | 3 | 4 | 5 | 6 | 7 |
| 8. I'm willing to donate a kidney to a second-degree relative  (uncle/aunt/cousin/  grandparent/  grandchild) | 1 | 2 | 3 | 4 | 5 | 6 | 7 |
| 9. I'm willing to donate kidney to a distant relative or friend. | 1 | 2 | 3 | 4 | 5 | 6 | 7 |
| 10. I'm willing to donate a kidney only to someone I know personally. | 1 | 2 | 3 | 4 | 5 | 6 | 7 |
| 11. I'm willing to donate a kidney only to someone from my own people. | 1 | 2 | 3 | 4 | 5 | 6 | 7 |
| 12. I'm willing to donate a kidney to anyone in need, regardless of religion, race, or gender | 1 | 2 | 3 | 4 | 5 | 6 | 7 |

1. **If you are not interested in donating a kidney while still alive, please mark the most appropriate answer in relation to the following statements according to the following scale:**

| Description | 7  Definitely  100% | 6  To a very great extent | 5  To a great extent | 4  To a moderate extent | 3  To a  small  extent | 2  To a very  small  extent | 1  Not at all |
| --- | --- | --- | --- | --- | --- | --- | --- |
| 16. I don't want to be left with only one kidney because it reduces my resilience and impairs my health. |  |  |  |  |  |  |  |
| 17. I don't trust the doctor and the medical team. |  |  |  |  |  |  |  |
| 18. I need time to think and study the kidney transplant’s medical procedure. |  |  |  |  |  |  |  |
| 19. I am not interested in donating a kidney for religious considerations |  |  |  |  |  |  |  |
| 20. I am not interested in donating a kidney because I am scared of the complications from doing so. |  |  |  |  |  |  |  |
| 21. I am not interested in donating a kidney because of fear of the surgery. |  |  |  |  |  |  |  |
| 22. I am not interested in donating a kidney because I don’t want my organ to be implanted into someone else’s body. |  |  |  |  |  |  |  |
| 23. I am not interested in donating a kidney because of objection by my family. |  |  |  |  |  |  |  |

1. Would you be willing to consider donating a kidney while you're alive for payment?

| Description | 7  Definitely  100% | 6  To a very great extent | 5  To a great extent | 4  To a moderate extent | 3  To a  small  extent | 2  To a very  small  extent | 1  Not at all |
| --- | --- | --- | --- | --- | --- | --- | --- |

7. What is the lowest (minimum) amount that would make you want to consider donating a kidney?

I am not willing to donate a kidney for payment.

1. $0–$3063.81^2^
2. $3064.12–$6127.63
3. $6127.93–$9191.44
4. $9191.75–$12255.26
5. $12255.56–$15319.07
6. $15319.38–$18382.88
7. $18383.19–$21446.70
8. $21447.00–$24510.51
9. $24510.82–$27574.33
10. $27574.63–$30638.
11. More than $ 30638. State the amount ____

8. Would you be willing to consider donating a kidney without receiving payment (altruistically) after your death?

| Description | 7  Definitely  100% | 6  To a very great extent | 5  To a great extent | 4  To a moderate extent | 3  To a  small  extent | 2  To a very  small  extent | 1  Not at all |
| --- | --- | --- | --- | --- | --- | --- | --- |

9. Would you be willing to consider donating a kidney **after your death** in exchange for payment to a family member?

| Description | 7  Definitely  100% | 6  To a very great extent | 5  To a great extent | 4  To a moderate extent | 3  To a  small  extent | 2  To a very  small  extent | 1  Not at all |
| --- | --- | --- | --- | --- | --- | --- | --- |

1. Would you be willing to consider donating a kidney of one of your first-degree relatives (child/brother/spouse/mother/father) **after their death** for payment?

| Description | 7  Definitely  100% | 6  To a very great extent | 5  To a great extent | 4  To a moderate extent | 3  To a  small  extent | 2  To a very  small  extent | 1  Not at all |
| --- | --- | --- | --- | --- | --- | --- | --- |

1. What is the lowest (minimum) amount that would make you consider donating your kidney **after your death?**

I am not willing to donate a kidney for payment.

1. $0–$3063.81^2^
2. $3064.12–$6127.63
3. $6127.93–$9191.44
4. $9191.75–$12255.26
5. $12255.56–$15319.07
6. $15319.38–$18382.88
7. $18383.19–$21446.70
8. $21447.00–$24510.51
9. $24510.82–$27574.33
10. $27574.63–$30638.
11. More than $ 30638. State the amount ____
12. What is the lowest (minimum) amount that would make you consider donating a kidney of one of your first-degree relatives (child/brother/spouse/mother/father) **after their death**?

I am not willing to donate a kidney of any of my relatives for payment after their death.

I am willing to donate a kidney of a relative after their death without receiving any payment.

1. $0–$3063.81^2^
2. $3064.12–$6127.63
3. $6127.93–$9191.44
4. $9191.75–$12255.26
5. $12255.56–$15319.07
6. $15319.38–$18382.88
7. $18383.19–$21446.70
8. $21447.00–$24510.51
9. $24510.82–$27574.33
10. $27574.63–$30638.
11. More than $ 30638. State the amount ____

1. In Israel, religious observance is a demographic factor that is used widely as a way for people to define themselves regarding their beliefs and practices. This is relevant when dealing with matters of organ transplantation which are regulated and circumscribed by religious law and doctrine. [↑](#footnote-ref-1)
2. The term 'traditional' covers a wide range of ideologies and levels of observance, and is based on self-definition. [↑](#footnote-ref-2)
3. The term 'religious' refers to those who follow the traditional religion. [↑](#footnote-ref-3)
4. The term 'secular' is not strictly defined, and it can mean either "not religious" or "convinced atheists". [↑](#footnote-ref-4)
